# Humor Therapy: Relieving Chronic Pain and Enhancing Happiness for Older Adults

**DOI:** 10.4061/2010/343574

**Published:** 2010-06-28

**Authors:** Mimi M. Y. Tse, Anna P. K. Lo, Tracy L. Y. Cheng, Eva K. K. Chan, Annie H. Y. Chan, Helena S. W. Chung

**Affiliations:** ^1^School of Nursing, The Hong Kong Polytechnic University, Kowloon, Hong Kong; ^2^Department of Medicine, Queen Elizabeth Hospital, Hong Kong; ^3^Department of Medicine, Pamela Youde Nethersole Eastern Hospital, Hong Kong; ^4^Department of Surgery, Queen Elizabeth Hospital, Hong Kong; ^5^Department of Medicine, Tseng Kwan O Hospital, Hong Kong; ^6^Department of Medicine, Tuen Mum Hospital, Hong Kong

## Abstract

The present study examined the effectiveness of a humor therapy program in relieving chronic pain, enhancing happiness and life satisfaction, and reducing loneliness among older persons with chronic pain. It was a quasiexperimental pretest-posttest controlled design. Older persons in a nursing home were invited to join an 8-week humor therapy program (experimental group), while those in another nursing home were treated as a control group and were not offered the program. There were 36 older people in the experimental group and 34 in the control group. Upon completion of the humor therapy program, there were significant decreases in pain and perception of loneliness, and significant increases in happiness and life satisfaction for the experimental group, but not for the control group. The use of humor therapy appears to be an effective nonpharmacological intervention. Nurses and other healthcare professionals could incorporate humor in caring for their patients.

## 1. Introduction

In light of the physiological changes associated with aging, older people are more susceptible to diseases of various kinds [1]. Indeed, most age-related diseases and illnesses bring chronic pain and disability. The reporting of any pain is common in the older adult population, with estimates of 55%–66% [2]. The prevalence of chronic pain is 25%–50% for community-dwelling older people [3, 4]. Pain is also common in nursing homes, with 45%–80% of nursing home residents suffering from substantial pain [5, 6]. Chronic pain is regarded as pain that persists past the normal time of healing [7]. Three months is the most convenient point of division between acute and chronic pain for nonmalignant pain [8]. 

Chronic pain is common in later life and is associated with negative mood states and life satisfaction [9]. Many older people accept pain as part of their life and do not seek help until it becomes severe and unbearable [9, 10]. The consequences of chronic pain among older people are considerable and include loneliness, social isolation, depression, impaired functional mobility and ambulation, and increased healthcare utilization and costs [11]. They tend to become isolated and unwilling to go out and meet friends and family members; also, they are less likely to express their feelings and thus more likely to become lonely. This may create misunderstandings and even interpersonal conflicts among family members and friends who are caring for them [12]. Indeed, these negative impacts of chronic pain are more disturbing for older persons in residential care. 

The management of chronic pain is generally inadequate. Physicians are often reluctant to prescribe adequate analgesic treatment for fear of inducing drug addiction [13]. It was found that over 50% of analgesic medications were ordered on an “as needed” basis [14]. This places responsibility for pain relief with the sufferer, in that they must request it. Furthermore, the treatment request is likely to be made only when the pain has reached the patient's tolerance limit. This limits the success of this strategy, because older people generally accept pain as part of growing old, and often fail to ask for pain relief despite experiencing severe pain [9, 15]. In this regard, the use of nonprescription, preferably nonpharmacological, pain relief measures is appealing to reduce the disability and distress associated with chronic pain. 

Nonpharmacological pain management strategies encompass a broad range of interventions and physical modalities, including education programs, cognitive-behavioral therapy, exercise programs, acupuncture, transcutaneous nerve stimulation, chiropractic, heat, cold, massage and relaxation [16]. The cognitive-behavioral approach to pain management focuses on the role of cognitive factors and their relationship to pain perception. It is reasonable to suggest that modification of cognition may be effective in altering the pain experience [17, 18]. Cognitive-behavioral strategies for pain management include hypnosis, relaxation with guided imagery, distraction, and the use of support groups [19]. Distraction is one of the important uses of cognitive-behavioral techniques to relieve pain, as suggested by the gate control theory [20]. And humor is one of the distraction techniques used in pain control. 

The gate control theory [20] describes three dimensions of pain: sensory-discriminative, motivational-affective, and cognitive control. The transmission of nerve impulses to spinal cord transmission cells is modulated by a spinal gating mechanism in the dorsal horn. The spinal gating mechanism is influenced by the amount of activity in large-diameter and small-diameter fibers carrying nerve impulses. Activity in the large fibers tends to inhibit transmission, whereas small-fiber activity tends to facilitate transmission. Likewise, the spinal gating mechanism is influenced by nerve impulses that descend from the brain. According to Melzack, selective cognitive processes are activated by a specialized system of large-diameter fibers and have the property of modulating the spinal gating mechanism by the descending fibers. Experimental and clinical studies support the concept of the transmission of pain as being modulated by afferent input from the periphery, descending inhibitory systems, and psychological factors.

In The Oxford English Dictionary, humor is defined as “that quality of action, speech, or writing which excites amusement; oddity, jocularity, facetiousness, comicality, fun” [21]. Humor involves cognitive, emotional, behavioral, psychophysiological, and social aspects [22]. Humor can refer to a stimulus such as a comedy film, a mental process such as perception, or a response such as laughter and exhilaration. Indeed, laughter is the most common behavioral expression of a humorous experience [23]. Humor and laughter are typically associated with a pleasant emotional feeling [24]. 

Humor has been shown to increase lung capacity, strengthen abdominal muscles, and increase immunoglobulin A, which is one of the major antibodies produced by the immune system [25, 26]. Humor causes reductions in cortisol, growth hormones, and epinephrine [27]. Following laughter or other humorous encounters, natural killer cell activity, immunoglobulin G and immunoglobulin M levels increase for as long as 12 hours [27, 28], and these evaluations bring about beneficial health outcomes. The use of humor consistently results in improvements in pain thresholds [29].

Humor also leads to the release of endorphins in the brain, which help to control pain [30]. In a laboratory study of pain tolerance using cold pressor stimulation, participants in the humor group had a significant increase in pain tolerance as compared to the other groups [31]. Pain management together with humor was found to be more effective than pain management alone [32]. Qualitative findings have also supported the effectiveness of humor in patient care [33, 34]. 

However, older people may not be able to receive sufficient training and education in cognitive therapies in pain relief and humor therapy in particular. In light of the inadequate management of chronic pain situations among older persons in residential care, and the potential therapeutic effects of humor, the present study proposed a humor therapy program for older people in order to relieve their chronic pain and enhance their psychological well-being. The objectives were to examine the effectiveness of humor therapy in relieving chronic pain, enhancing happiness and life satisfaction, and reducing loneliness among older people with chronic pain. 

## 2. Methods

### 2.1. Design & Sample

This research was a quasi-experimental pre- and posttest control group design. After gaining approval from the university's Ethics Committee, an organization operating residential care homes for older people was approached and invited to participate in the study. Under this organization, two nursing homes were randomly selected, one as the experimental group (receiving humor therapy) and the other as the control group (without humor therapy). These two nursing homes were similar in nature, running under the same organization, with similar staff to client ratios. In addition, resources, spacing and quality of services were similar in these homes. 

Posters were displayed in the function rooms and hallways of the nursing homes to inform and invite residents to join the study; also, nursing home staff recommended that the research team approach potential residents that fit the inclusion criteria. Written consent was obtained from all participants. Inclusion criteria for the participants included being able to communicate in Cantonese, being cognitively intact based on the AMT (score of ≥8) [35], having experienced pain in the previous three months, and being willing to participate in the entire humor therapy program. By contrast, those who were cognitively disabled, had mental disorders or were completely blind or deaf were excluded from this study. 

**Figure 1:**
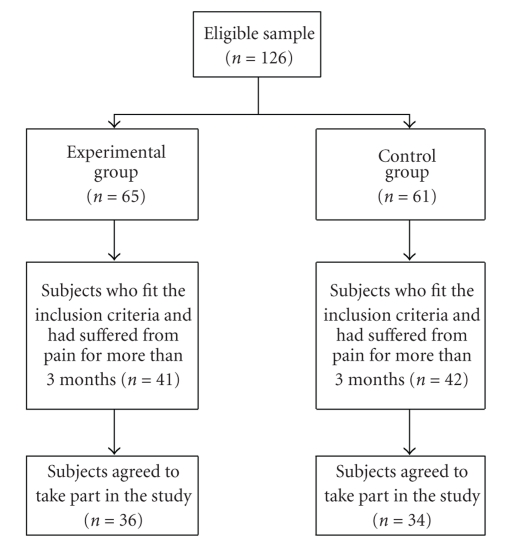
Summary of the workflow of the study.

In the experimental group, 65 older persons were approached by the research team and invited to join the study. There were 41 who fit the inclusion criteria and had suffered from pain for more than three months, and 36 of them agreed to take part in our study. As for the control group, 61 older persons were approached, of whom 42 had suffered from pain for more than three months and fit all the inclusion criteria. Of these, 34 agreed to join the study. This procedure is illustrated by the consort diagram in Figure 1.

### 2.2. Humor Therapy Program

Humor therapy was carried out in the multifunction room of the nursing home. It was an 8-week program involving one hour per week. The overall atmosphere was relaxed and cheerful during all the humor therapy sessions. 

In the first week, each participant would receive a portfolio called “My Happy Collection”. The research team would work with the participants to design and make entries in the portfolios, with funny books and photos, jokes, funny audio tapes and videos, comedy clips and cartoons, and funny and interesting news clips, articles, stories and reflections. Their portfolios were reviewed every week, and any difficulties and happiness in making the portfolios were shared. 

In the following weeks, humor therapy was carried out by the research team. From week 2 to week 8, each session started with a joke of the day and the reading of funny jokes and stories; lectures on humor research were then given. Participants in the therapy group were also shown how to give higher priority to humor in their everyday lives, laughing exercises and games, sharing of their own funny stories, magic shows, and hot tips to stimulate humor and joy. 

At the end of the 8th week, portfolios were shared among all participants. Post-test questionnaires were also collected, and participants in the experimental group were invited for an interview to share their experience of the humor therapy. 

### 2.3. Procedures and Instruments

Demographic data including gender, age, previous health history, and time spent in nursing homes were collected for all participants. Also, perception of pain (assessed using the Cantonese Verbal Rating Scales) [36–38], and psychological parameters including happiness (assessed using the Subjective Happiness Scale [39]), loneliness (assessed using the Revised UCLA Loneliness Scale [40]), and life satisfaction (assessed using the Revised Life Satisfaction Index-A scale [41]) were examined before week 1 and after the 8-week humor therapy program for all participants. 

Pain was measured by the Cantonese Verbal Rating Scales [36–38] and consisted of a series of words commonly used to describe pain. The scale has 11 descriptors: 0 = no pain, 1 = very mild pain, 2 = uncomfortably painful, 3 = tolerable pain, 4 = distressingly painful, 5 = very distressingly painful, 6 = intense pain, 7 = very intense pain, 8 = utterly horrible pain, 9 = excruciatingly unbearable, and 10 = unimaginably unspeakable pain. Participants were instructed to read the words and choose the option that best described the pain in their experience. The reliability and validity of the pain scale in the Cantonese VRS have been established previously [36–38].

Happiness was measured by The Subjective Happiness Scale [39], which consists of a 4-item measure of global subjective happiness. Items are rated on a 7-point Likert scale with different descriptors for each item. The Cronbach's alpha was 0.79 to 0.94 [39]. The test-retest reliability ranged from 0.55 to 0.90. Two items asked participants to characterize themselves using both absolute ratings and ratings relative to peers, while the other two items offered brief descriptions of happy and unhappy individuals and asked participants the extent to which each characterization described them. The total range of the scores was 4–28, with higher scores reflecting greater happiness. Permission was obtained from Professors Lyubomirsky & Lepper for the translation and use of the SHS in the present study. The SHS used only 4 short, simple questions, and every effort was made to render the Chinese translation of the SHS easily understandable and applicable to the Chinese elderly in the present study. 

The Revised UCLA Loneliness Scale is a standard scale for the measurement of loneliness [40]. In version 3, there are 20 items, including 9 positively and 11 negatively worded items. Interviewees are asked to rate how frequently they feel as described, from “never” to “often”. Each of the 20 items is rated on a scale of 1 (never), 2 (rarely), 3 (sometimes), and 4 (often). After reverse scoring appropriate items, loneliness scores were calculated by summing all items. The range of possible scores was 20 to 80, with higher scores signifying greater loneliness. Scores from 30 to 40 are considered a normal experience of loneliness, while scores above 60 indicate that a person is experiencing severe loneliness. Reliability testing indicates that the internal consistency of this scale has a Cronbach's alpha ranging from 0.89 to 0.94 and the test-retest reliability is 0.73. A Chinese version of the Revised UCLA Loneliness Scale was validated and used, and the Cronbach's alpha of the Chinese UCLA Loneliness Scale was 0.90 [42].

The Life Satisfaction Index-A [43] form scale consists of 18 questions related to five different components: zest, resolution and fortitude, congruence between desired and achieved goals, positive self-concept and mood tone. Items scored 1 point for agree and 0 for disagree. Reverse scoring appropriate items provided a range of scores from 0 to 18, with the highest scores indicating the greatest satisfaction. A Chinese version of the Life Satisfaction Index-A form was used, with the Cronbach's alpha 0.7 for reliability and split half value 0.62 for internal consistency [41].

### 2.4. Data Analysis

Several statistical methods were used in data analysis. Descriptive statistical analysis of the quantitative data was conducted using the Statistical Package for the Social Sciences (SPSS) version 13.0. The Chi-square and Mann-Whitney U tests were used to determine any differences between the experimental and control groups, while the Wilcoxon Signed Ranks Test was used to examine differences within groups over two occasions. The Friedman test was used to detect changes in the happiness and pain scores throughout the 8-week humor therapy in the experimental group. A *P*-value <.05 was considered statistically significant. 

## 3. Results

### 3.1. Demographic Data

**Figure 2:**
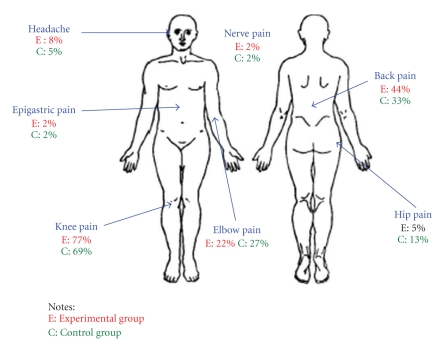
Location of pain.

Of the 70 older people who participated in the study, 36 were in the experimental group and 34 in the control group. There were 38 females and 32 males, and their ages ranged from 65 to 95 years, with the median age ranging from 80 to 89. The mean age was 78.25 for the experimental group and 79.38 for the control group.  Table 1 shows the demographic data and Figure 2 shows the locations of pain for participants in both groups. 

Most of the participants had been in a nursing home for 1–3 years. No significant differences were found in the demographic characteristics of sex, age, time spent in a nursing home, past health history, pain scores, situation of pain (including pain scores at the baseline), and use of medications between older people in the experimental group and the controls (*P* > .05). In terms of past health history, it was found that more participants in the control group had DM than in the experimental group. In spite of this, these participants' DM condition had been stable and controlled by dietary interventions and or medication, thus the presence of DM was not considered to affect their performance and enjoyment of the humor therapy. Other than their past health history, participants in the experimental and control groups were similar in terms of their demographic characteristics. 

### 3.2. Pain and Psychological Parameters for Older People

#### 3.2.1. Baseline (week 1) for Experimental and Control Groups

Pain scores of 5.19 and 5.50 in the experimental and control groups indicated medium pain intensity; the location of pain was mainly the knee and back, and it was musculoskeletal in nature. Because participants had experienced pain over the previous three months, it was regarded as chronic pain. There were no significant differences in the pain scores and psychological parameters of participants between the experimental and control groups (*P* > .05) at the baseline (week 1) (Table 1) As such, both groups were found to be identical in their pain situation, as well as in happiness, loneliness and life satisfaction. 

#### 3.2.2. Postintervention (week 8) for the Experimental and Control Groups

There were significant improvements in the pain scores and all psychological parameters for the experimental group postintervention (in week 8) as compared to the baseline (week 1) (*P* < .05) (Table 2), but no similar improvements in the control group (*P* > .05). In addition, there were significant differences between the experimental and control groups in terms of reduction of pain scores and increase in happiness and life satisfaction in the postintervention phase (*P* < .05), except for the loneliness scores (*P* > .05). As such, there was a significant reduction in pain scores from 5.19 ± 2.12 to 3.22 ± 1.48, and a reduction in loneliness from 42.50 ± 8.25 to 39.44 ± 7.96 after the humor therapy intervention, as well as significant increases in happiness from 16.19 ± 5.14 to 23.03 ± 3.40 and life satisfaction from 10.50 ± 2.88 to 12.67 ± 2.22 for participants in the experimental group. However, there was no such significant reduction in pain or increase in happiness and life satisfaction for participants in the control group.

#### 3.2.3. Pain and Happiness for Participants in the Experimental Group

**Figure 3:**
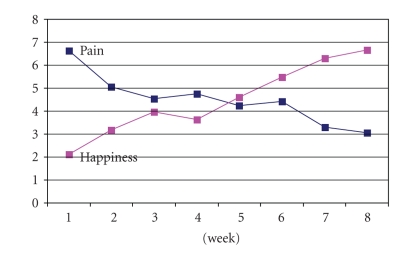
Pain and happiness scores over the 8-week humor therapy program. ^#^The Friedman Test was used. *A *P*-value of ≦ .05 was considered statistically significant.

The Friedman test was used to test the changes in the happiness and pain scores across the 8-week humor therapy program.  Figure 3 shows the decreasing trend in the mean rank pain scores and the increase in the mean rank happiness scores; these changes were statistically significant (*P* < .05). 

## 4. Discussion

The present study demonstrated the therapeutic effects of humor therapy in reducing pain and loneliness and enhancing happiness and life satisfaction among older people with chronic pain living in nursing homes. 

The prevalence and impact of chronic pain continue to increase; as such, the day-to-day management of chronic pain presents a major challenge. In the present study, the majority of older persons (61% to 69%; experimental to control groups) stated that they had experienced pain over the previous three months. The pain intensity was found to be high, with a mean pain intensity of more than five on a 10-point numeric pain scale. These findings were consistent with the literature, suggesting that older residents in nursing homes were in pain [5, 6, 10]. 

In the present study, the onset of pain for the elderly could take place at any time (19% to 24%), during sleep (28% to 38%), on waking (14% to 12%) and while performing exercise (44% to 68%). These findings illustrate the impact of chronic pain on the quality of life of older people. One of the primary causes of a sedentary lifestyle is chronic pain, and this is more common among the elderly [44]. As a result of pain, it is difficult for older people to perform regular exercise and engage in social events. Indeed, chronic pain limits physical and functional mobility and ambulation, leading to muscle atrophy and causing falls and injury [45]. 

Unfortunately, the use of medications and nonpharmacological methods as pain relief has been woefully inadequate. Only 32% to 39% of our participants took pain medications to relieve pain. These findings are consistent with the literature regarding older people's actions to seek medications [14, 15]. 

It is found that older people are reluctant to request pain relief, attempting to endure pain as a “normal” part of aging and wishing to avoid being labeled as “complainers” [5]. It may be difficult to encourage patients to demand more complete relief of pain. To this end, nonpharmacological interventions can be very effective for all types and intensity of pain, and are thus recommended when used concurrently with pharmacological interventions in the treatment of pain [46]. 

It is also worrying to find that participants in both the experimental and control groups had low happiness and life satisfaction scores, and also moderately high scores in loneliness at the baseline. The maximum score for happiness was 28, with higher scores reflecting greater happiness; as such, the happiness scores of around 16 for both experimental and control groups indicate a relatively low level of happiness. Likewise, the maximum score for life satisfaction was 18, and scores of around 10 for participants in both groups indicated relatively low satisfaction in life. It is also worrying to find that the loneliness scores were around 42 for older people in both groups, indicating experiences of loneliness. These findings were consistent with the literature suggesting that residents in nursing homes experienced relational losses including loss of spouse, relatives and friends, and that these losses may lead to social isolation and loneliness [47]. As such, nursing home residents are found to be socially isolated and feeling very lonely [48, 49]. 

The cognitive therapy for managing pain emphasizes the role of cognitive, affective and behavioral factors in the development and maintenance of chronic pain (Castro-Lopes, 2008). Cognitive therapy reduces feelings of helplessness and lack of control, and establishes a sense of control over pain. As such, older persons with chronic pain learn to use various techniques to effectively deal with episodes of pain. In this regard, the use of humor therapy appears to be an effective cognitive, nonpharmacological intervention in chronic pain management, enhancing happiness and life satisfaction and reducing loneliness for older people. The needs of older people include pride, maintaining dignity, social contacts, and activity [50]. Upon completion of the humor therapy, participants in the experimental group had a significant reduction in pain sensation and felt less loneliness, and they were happier and more satisfied with their lives. Participants did express happiness and laughter. They shared and laughed at their own funny stories, and were able to appreciate life situations from a humorous perspective. They also felt like members of a team when engaged in the laughing and humor activities. There was no significant change in the pain score, happiness and life satisfaction among older persons in the control group. 

Indeed, the use of humor gives us permission to laugh and to relax [51]. Materials for creating a humorous environment include funny movies, audio and videotapes of humorous songs, books, and games for patients of every age. The use of the joke of the day on the internet, jokes and funny stories all add value in helping patients who would otherwise have very little or nothing to laugh about [52]. 

A very common pain-relieving cognitive strategy used by older people is distraction [53]. Distraction is achieved by asking subjects to attend to another sensory modality, such as an auditory, visual and tactile stimulus [54, 55]. In this regard, the older people in the nursing home could, for example, be encouraged to watch funny videos or listen to jokes or funny music as a means to involve more sensory modalities and so help in their coping with pain. 

Nevertheless, it is noted that the older persons in the present study may have given “socially desirable” responses in the posttest. The fact that the research team visited the nursing home on a regular basis to lead the humor therapy program made them appear to the older persons as being of good intention. In this way, the positive results reported by the older persons may have been the effect of a therapeutic relationship, and not due to the humor therapy program [56]. This constitutes a limitation to the present study. As such, the use of a control group with social activities and one with social activities and humor therapy program is recommended in any further study. Also, it is suggested that happiness and pain scores be collected across the 8-week program for both experimental and control groups in any further study. Further, raters were not blind to the study. Raters administered the pre- and posttest assessments and led the humor therapy program in both the control and experimental groups, yet the research team reinforced the importance of not being biased. In future studies, different raters should be used for the pre- and posttest assessments and for the intervention. The present study used a convenience sampling method, which may constitute a limitation to the study. Another is the use of a translated psychosocial measure for older adults in nursing homes. Further studies are needed to validate the psychosocial measure in this population. 

To sum up, given the positive effects of humor therapy in the experimental group, the research team reported these findings to the in-charge of both nursing homes. As a result, the control group was visited by the research team at the end of the study and humor therapy activities were performed. 

## 5. Conclusions & Relevance to Clinical Practice

It is noted that people aged 80 and over will continue to postpone disabling health problems as long as they maintain a relatively vigorous level of intellectual, interpersonal and physical activity [57]. In light of the high prevalence of chronic pain and its impact on physical and psychological perspectives among older people, the use of humor therapy as a means of reducing pain and loneliness as well as increasing happiness and life satisfaction is very appealing. Nurses and other healthcare professionals can incorporate humor in caring for their patients. Telling a joke and encouraging clients to tell a funny story may have a therapeutic effect [58]. Asking patients to make a “My Happy Folder” is also a good way to involve and empower them in their own pain and symptom management. Regardless of their physical condition, patients need to allow themselves to be happy, to let humor play a greater role in their lives, and to enjoy life [34]. Using humor therapy is a good method of health maintenance, as suggested by the participants in the present study. 

## Figures and Tables

**Table 1:** Demographic data for participants of experimental and control groups.

	Experimental group (%) *n* = 36 mean (SD)	Control group (%) *n* = 34 mean (SD)	*P*-value^#^
Sex			.052

Female	15 (*42*)	23 (*68*)	
Male	21 (*58*)	11 (*32*)	

Age			*.365*

Mean	78.25	79.38	
Range	60–89	65–92	

Time spent in nursing home (years)			*.082*

1–3 years	15 (*42*)	20 (*59*)	
4–6 years	11 (*31*)	12 (*35*)	
7–9 years	6 (*17*)	2 (*6*)	
≥10 years	4 (*11*)	0 (*0*)	

Past health history (more than one can be selected)			

Stroke	5 (*14*)	2 (*6*)	*.264*
Hypertension	17 (*47*)	16 (*47*)	*.989*
DM	6 (*17*)	14 (*41*)	.045*
Cardiac disease	6 (*17*)	3 (*9*)	*.534*
Others	23 (*64*)	20 (*59*)	*.850*

Pain scores (baseline)			

Pain scores	5.19 ± 2.12	5.50 ± 1.88	.612

Situation of pain (more than one can be selected)			

Any time	7 (*19*)	8 (*24*)	*.677*
Sleeping	10 (*28*)	13 (*38*)	*.352*
Waking up	5 (*14*)	4 (*12*)	*.791*
Exercising	16 (*44*)	23 (*68*)	*.051*
Grooming	0 (0)	4 (12)	*.109 *
Others	4 (*11*)	0 (*0*)	*.137*
No	22 (*61*)	23 (*68*)	

Use of medications as pain relief			

Yes	14 (39)	11 (32)	*.748*
No	22 (61)	23 (68)	

^∧^Percentages may not add up to 100% because of rounding.

^#^Chi-square test was used.

*P-value of <.05 was considered statistically significant.

**Table 2:** Comparison of experimental & control groups: baseline (week 1) versus postintervention (week 8).

	Experimental group (*n* = 36)		*P*-value *β* ^2^	Control group (*n* = 34)		*P*-value *β* ^3^	*P*-value *β* ^1^	*P*-value *β* ^4^
	Baseline (week 1) Mean ± SD	Post interventio (week 8) Mean ± SD		Baseline (week 1) Mean ± SD	Post intervention (week 8) Mean ± SD			
Pain	5.19 ± 2.12	3.22 ± 1.48	0.000*	5.5 ± 1.88	5.18 ± 1.62	0.150	0.612	0.000*
Happiness	16.19 ± 5.14	23.03 ± 3.40	0.000*	16.82 ± 4.9	17.00 ± 4.65	0.958	0.628	0.000*
UCLA (Loneliness)	42.50 ± 8.25	39.44 ± 7.96	0.001*	43.29 ± 9.14	42.85 ± 8.92	0.291	0.629	0.096
Life Satisfaction	10.50 ± 2.88	12.67 ± 2.22	0.000*	10.12 ± 3.21	10.32 ± 2.76	0.470	0.736	0.001*

*β* ^1^ Experimental versus control group at baseline (week 1); (Mann-Whitney U Test).

*β* ^2^ Experimental group (baseline versus postintervention); (Wilcoxon Signed Ranks Test).

*β* ^3^ Control group (baseline versus postintervention); (Wilcoxon Signed Ranks Test).

*β* ^4^ Experimental group versus control group postintervention (week 8); (Mann-Whitney U Test).

*A *P*-value of <.05 was considered statistically significant.
